# The complete chloroplast genome sequence of *Syringa meyeri* (Oleaceae)

**DOI:** 10.1080/23802359.2025.2512866

**Published:** 2025-06-03

**Authors:** Shaoying Cheng, Jingrui Li, Hongxia Cui, Yuanyuan Li, Zhonghua Liu

**Affiliations:** a School of Biological Sciences and Technology, Beijing Forestry University, Beijing, China; b State Key Laboratory of Plant Diversity and Specialty Crops & Institute of Botany, Chinese Academy of Sciences, Beijing; c China National Botanical Garden, Beijing, China; d School of Landscape Architecture, Beijing Forestry University, Beijing, China

**Keywords:** Oleaceae, chloroplast genome, Syringa meyeri, phylogenetic analysis

## Abstract

*Syringa meyeri* C. K. Schneid. 1912 (Oleaceae) is a common dwarf ornamental lilac, native to Northeast China. In this study, the complete chloroplast genome of *S. meyeri* was assembled, exhibiting a typical quadripartite structure with a total length of 160,459 bp and a GC content of 37.9%. A total of 141 genes were annotated, including 96 protein-coding genes, 37 tRNA genes, and eight rRNA genes. Phylogenetic analysis of 19 cp genomes indicated that *S. meyeri* is most closely related to *S. pubescens* subsp. *microphylla.* These results provide essential genomic resources for future phylogenetic and evolutionary studies in *Syringa*.

## Introduction

Lilacs (*Syringa* L., Oleaceae) have long been used in gardening, essential oil production, and for medicinal purposes in both Europe and East Asia (Wang et al. 2022). Taxonomically, *Syringa* comprises five groups: *Series Syringa*, *Ser. Pinnatifoliae*, *Ser. Villosae*, *Ser. Pubescentes* and *Sect. Ligustrina*. S*er. Pubescentes* includes approximately one-third of all *Syringa* species (Chang and Qiu 1992), contributing to its considerable species diversity. The dwarf species *Syringa meyeri* C. K. Schneid. 1912 from *Ser. Pubescentes*, is native to Liaoning Province in Northeast China (Chang and Qiu 1992). Its compact size, coupled with its elegant flowers and pervasive fragrance, has made it a popular ornamental plant, widely cultivated in the northern regions of China (Gui et al. 2023). Despite its popularity, few studies on this species have been reported.

The chloroplast genome is a useful tool for phylogenetic analyses (Olofsson et al. 2019; Dupin et al. 2020). Approximately half of the cp genomes of *Syringa* have been sequenced (e.g. Olofsson et al. 2019; Zhang et al. 2019; Liu et al. 2020; Zhao et al. 2020; Yang et al. 2023). However, there remains a need to examine the chloroplast genome of this morphologically distinct dwarf species within the genus, although its sister species, the genome of *S. pubescens* subsp. *microphylla* from *Ser. Pubescentes*, has been sequenced (Van de Paer et al. 2018). In this study, we present the first complete assembly of the chloroplast genome of *S. meyeri* using next-generation sequencing technology, followed by a comprehensive analysis.

## Materials and methods

**Figure 1.**
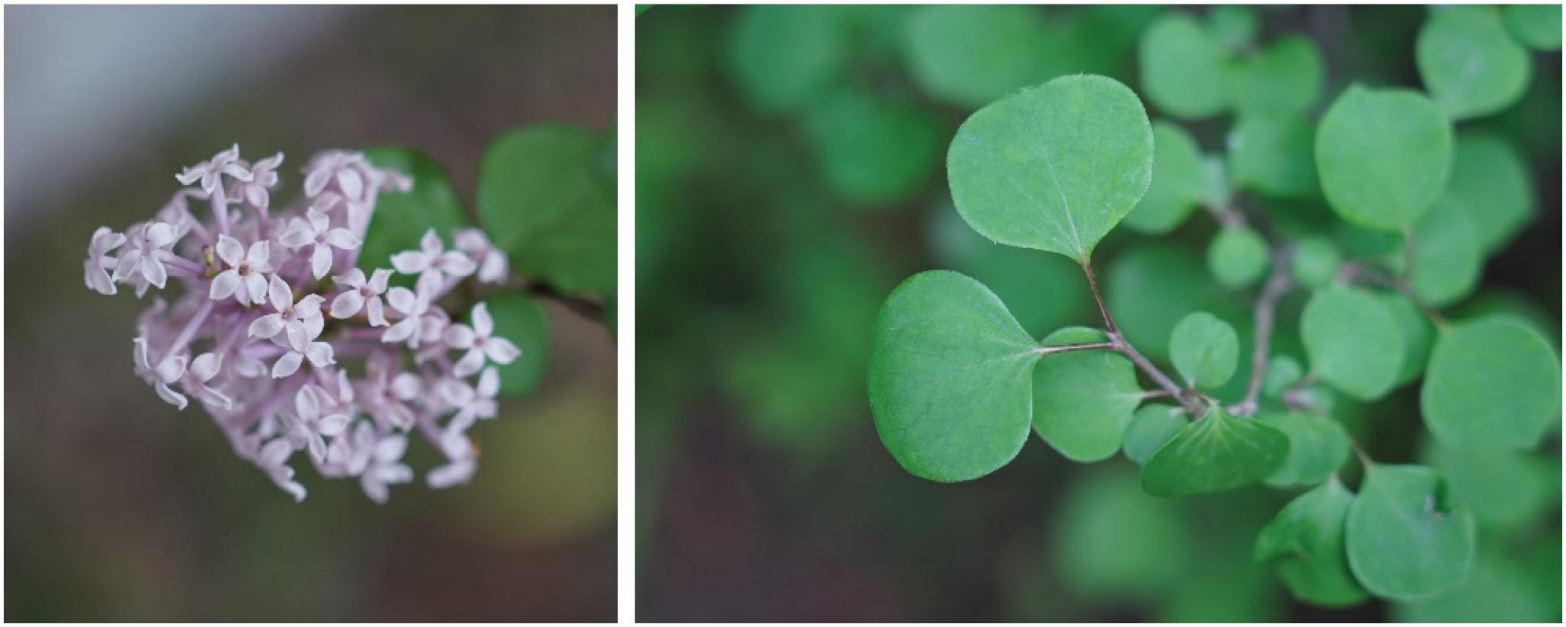
*Syringa meyeri* from the institute of botany, chinese academy of sciences (E 116°12’59’’, N 39°59’20’’), features light purple corollas with elliptical lobes that are apically beaked, along with suborbicular leaves bearing two pairs of lateral veins converging at the leaf base. Photographs were taken by shaoying cheng.

*meyeri* (Figure 1) was cultivated at the Institute of Botany, Chinese Academy of Sciences (E 116°12’59′’, N 39°59’20′’). The voucher specimen was deposited at the herbarium of the Institute of Botany, Chinese Academy of Sciences (Li Zexin pelizexin@ibcas.ac.cn, accession number: 2763202). Total genomic DNA was extracted from fresh leaves using the CTAB method and sequenced on the Illumina × ten platform. The per-base coverage depth of the *S. meyeri* chloroplast genome was generated using SAMtools v1.17 (Danecek et al. 2021) and Bowtie 2 v2.5.4 (Langmead and Salzberg 2012). The chloroplast genome was assembled with the GetOrganelle v1.7.5 (Jin et al. 2020). The assembled genome was annotated with CPGAVAS2 (Shi et al. 2019), using the chloroplast genome of *S. pubescens* subsp. *microphylla* (GenBank number: MT872641.1) as the reference sequence, and visualized using CPGView (Liu et al. 2012). The tool was also used to generate cis-spliced and trans-spliced genes. To explore the phylogenetic relationships of *S. meyeri*, we selected 26 species from the Oleaceae family and used three species of *Forsythia*, namely *Forsythia mandschurica* (NC_048504.1), *Forsythia suspensa* (MF579702.1), and *Forsythia mira* (NC_046065.1), as outgroups to construct a phylogenetic tree. With the exception of *S. meyeri*, the cp genomes of the other species were downloaded from the GenBank database. The chloroplast genome of *S. meyeri* was aligned with those of the other 26 species using MAFFT with default parameters (Katoh and Standley 2013). We used IQ-TREE v1.6.12 (Nguyen et al. 2015) to construct a maximum likelihood (ML) tree, with the optimal evolutionary model identified as TVM+F + I + G4, based on the Bayesian Information Criterion (BIC).

## Results

To assess the accuracy of the assembly, we evaluated the per-base coverage depth of the *S. meyeri* chloroplast genome. The coverage depth ranged from 22× to 2,612×, with a mean depth of 1,680×, confirming the reliability of the assembled genome (Figure S1).

**Figure 2.**
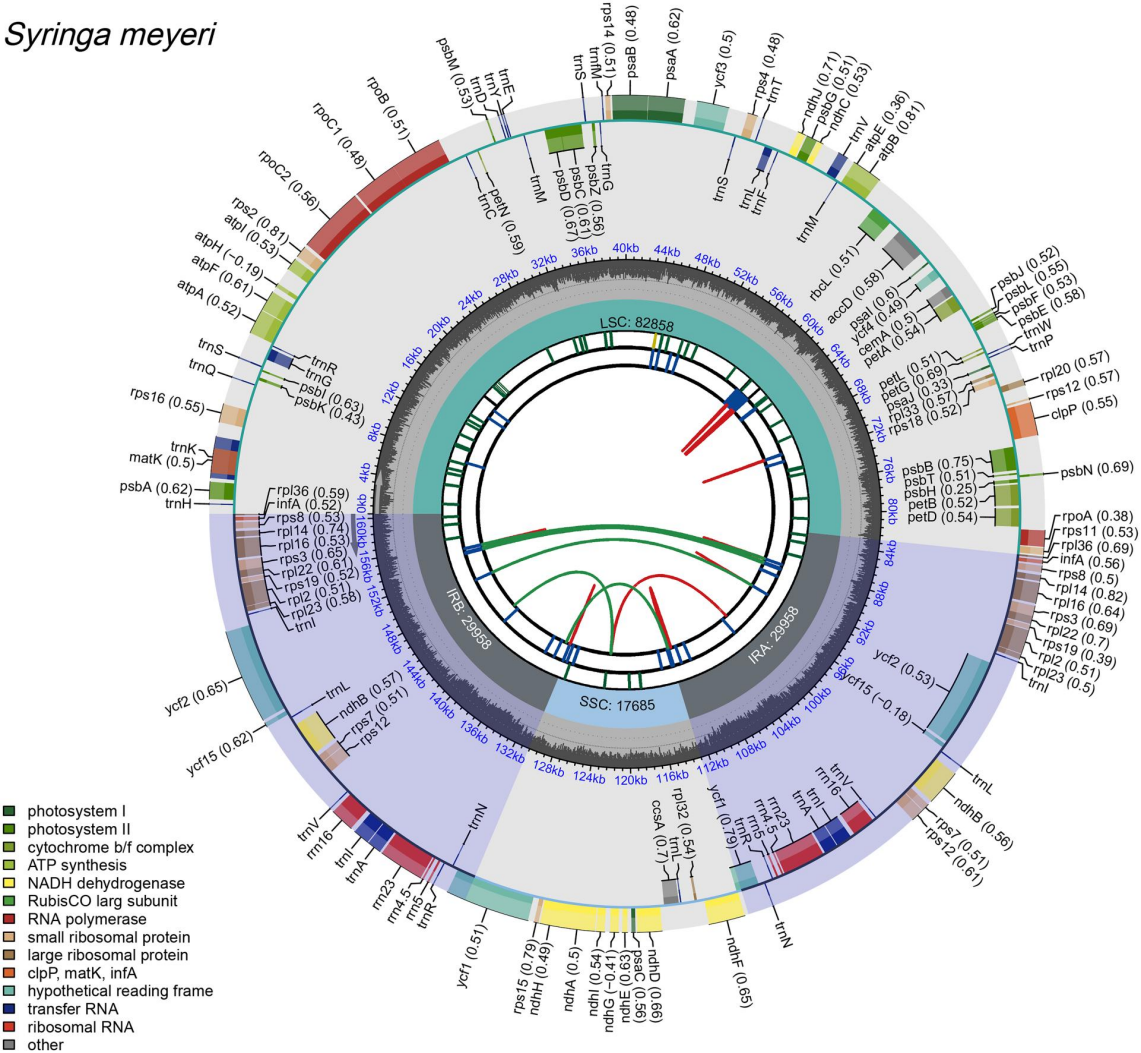
Annotated chloroplast genome map of *Syringa meyeri*. The first sequence track, radiating from the core, displays the forward and reverse repeats, which are indicated by green and red arcs. The second and third tracks show the tandem repeats and microsatellite sequences, each highlighted in distinct colors. The outermost track categorizes genes based on their functions, with the corresponding color scheme shown at the lower left corner.

The sequencing and assembly revealed that the length of the *S.meyeri* chloroplast genome is 122,586 bp, forming a circular structure with a typical quadripartite arrangement. This includes a pair of inverted repeats (IRs) of 29,958 bp separated by a large single-copy (LSC) region of 82,858 bp and a small single-copy (SSC) region of 17,685 bp (Figure 2). The GC content of the chloroplast genome is 37.9%. A total of 141 functional genes were annotated, including 96 protein-coding genes, 37 tRNA genes, and eight rRNA genes. Additionally, the following genes were found to contain introns: *rps*16, *atp*F, *rpo*C1, *pet*B, *pet*D, *rpl*16, *rpl*2, *ndh*B, *ndh*A, *trn*K-UUU, *trn*G-GCC, *trn*L-UAA, *trn*V-UAC, *trn*I-GAU, and t*rn*A-UGC each contain one intron, while *rps*12, *ycf*3 and *clp*P each contain two introns (Figure S2). The *rps*12 gene is a trans-spliced gene, with distinct exons located at different genomic positions, connected through a complex splicing process to form the mature mRNA (Figure S3).

**Figure 3.**
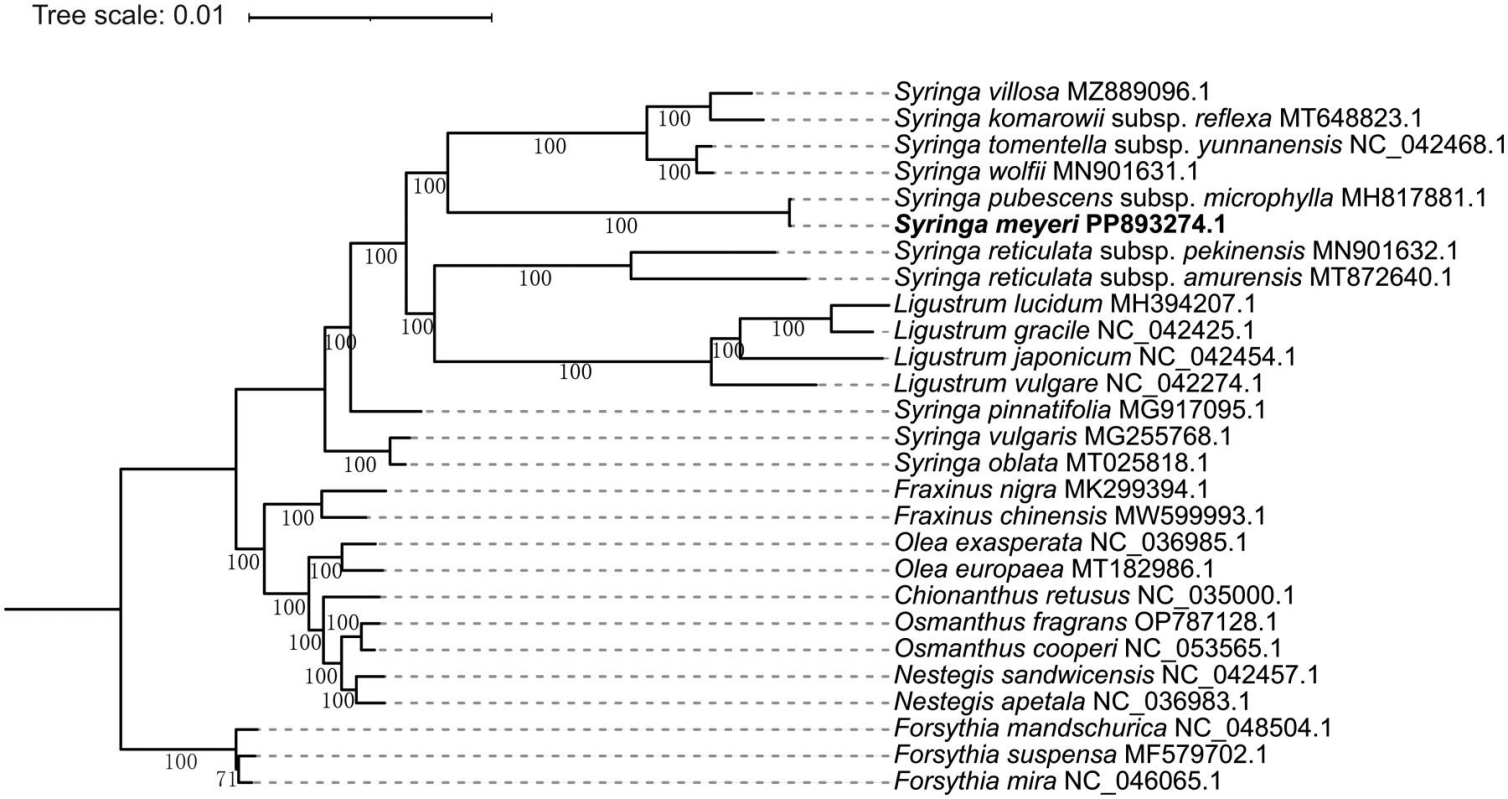
Maximum likelihood (ML) phylogenetic tree based on the protein-coding genes in the chloroplast genome of *Syringa meyeri* and 26 plastomes of the oleaceae family. The numbers above the branches represent the bootstrap values from 1000 rapid repetitions, and the legend indicates the scale of nucleotide substitutions. *Forsythia* species were used as outgroups in the phylogenetic tree. The sequences included in this tree were downloaded from the NCBI GenBank. Accession numbers are: *Syringa vulgaris* (MG255768.1) (unpublished), *syringa oblata* (MT025818.1) (Zhao et al. 2020), *syringa pinnatifolia* (MG917095.1) (Zhang et al. 2019), *syringa reticulata* subsp. *pekinensis* (MN901632.1) (Wang et al. 2020), *syringa reticulata* subsp. *amurensis* (MT872640.1) (unpublished), *syringa wolfii* (MN901631.1) (Liu et al. 2020), *syringa villosa* (MZ889096.1) (unpublished), *syringa komarowii* subsp. *reflexa* (MT648823.1) (unpublished), *syringa pubescens* subsp. *microphylla* (MH817881.1), *syringa tomentella* subsp. *yunnanensis* (NC_042468.1), *ligustrum gracile* (NC_042425.1), *ligustrum japonicum* (NC_042454.1), *ligustrum vulgare* (NC_042274.1), *fraxinus nigra* (MK299394.1), *nestegis sandwicensis* (NC_042457.1), *forsythia mandschurica* (NC_048504.1) (Olofsson et al. 2019), *ligustrum lucidum* (MH394207.1) (Wang et al. 2018), *fraxinus chinensis* (MW599993.1) (unpublished), *olea exasperata* (NC_036985.1) (unpublished), *Olea europaea* (MT182986.1) (Niu et al. 2020), *osmanthus fragrans* (OP787128.1) (unpublished), *osmanthus cooperi* (NC_053565.1) (Wang et al. 2019), *nestegis apetala* (NC_036983.1) (unpublished), *chionanthus retusus* (NC_035000.1) (He et al. 2017), *forsythia suspensa* (MF579702.1) (Wang et al. 2017), and *forsythia mira* (NC_046065.1) (Gao et al. 2019).

The phylogenetic tree constructed using the complete chloroplast sequences from *Syringa* and other genera within the Oleaceae family reveals that *Syringa meyeri* and *Syringa pubescens* subsp. *microphylla* cluster together as sister taxa. This suggests a close phylogenetic relationship between the two species, with 100% support for this clade (Figure 3).

## Discussion and conclusions

Phylogenetic trees constructed using complete plastid sequences of *S. meyeri* and other species of *Syringa*, along with other genera from the Oleaceae family, indicate that *S. meyeri* shares the closest relationship with *S. pubescens* subsp. *microphylla*, which is in agreement with the phylogenetic tree based on the nuclear genome (Wang et al. 2022). *Ligustrum* forms a monophyletic group nested within *Syringa*, identifying *Syringa* as a non-monophyletic genus. These findings are consistent with previous studies on the phylogenetic relationships within Oleaceae and *Syringa* (Dupin et al. 2020; Dupin et al. 2024; Li et al. 2002; Olofsson et al. 2019; Wang et al. 2025).

In this study, we present the first assembly and annotation of the chloroplast genome sequence of *S. meyeri*, which spans 160,459 bp in length, has a typical tetrameric structure, and is closely related to *S. pubescens* subsp. *microphylla*. This work lays the groundwork for future phylogenetic investigations of *Syringa*.

## Supplementary Material

Supplementary Material Figure S3.tif

Supplementary Material Figure S2.tif

Supplementary Material Figure S1.tif

## Data Availability

The genome sequence data that support the findings of this study are openly available in GenBank of NCBI at (https://www.ncbi.nlm.nih.gov/) under the accession no. PP893274. The associated BioProject, SRA, and Bio-Sample numbers are PRJNA1158012, SRR30669901, and SAMN43530668 respectively.
