# Supplementary figures and images for: The complete chloroplast genome sequence of Syringa meyeri (Oleaceae)
Source: Mitochondrial DNA B Resour. 2025 Jun 3;10(7):543–7. doi: 10.1080/23802359.2025.2512866 (PMC12135084; doi:10.1080/23802359.2025.2512866)

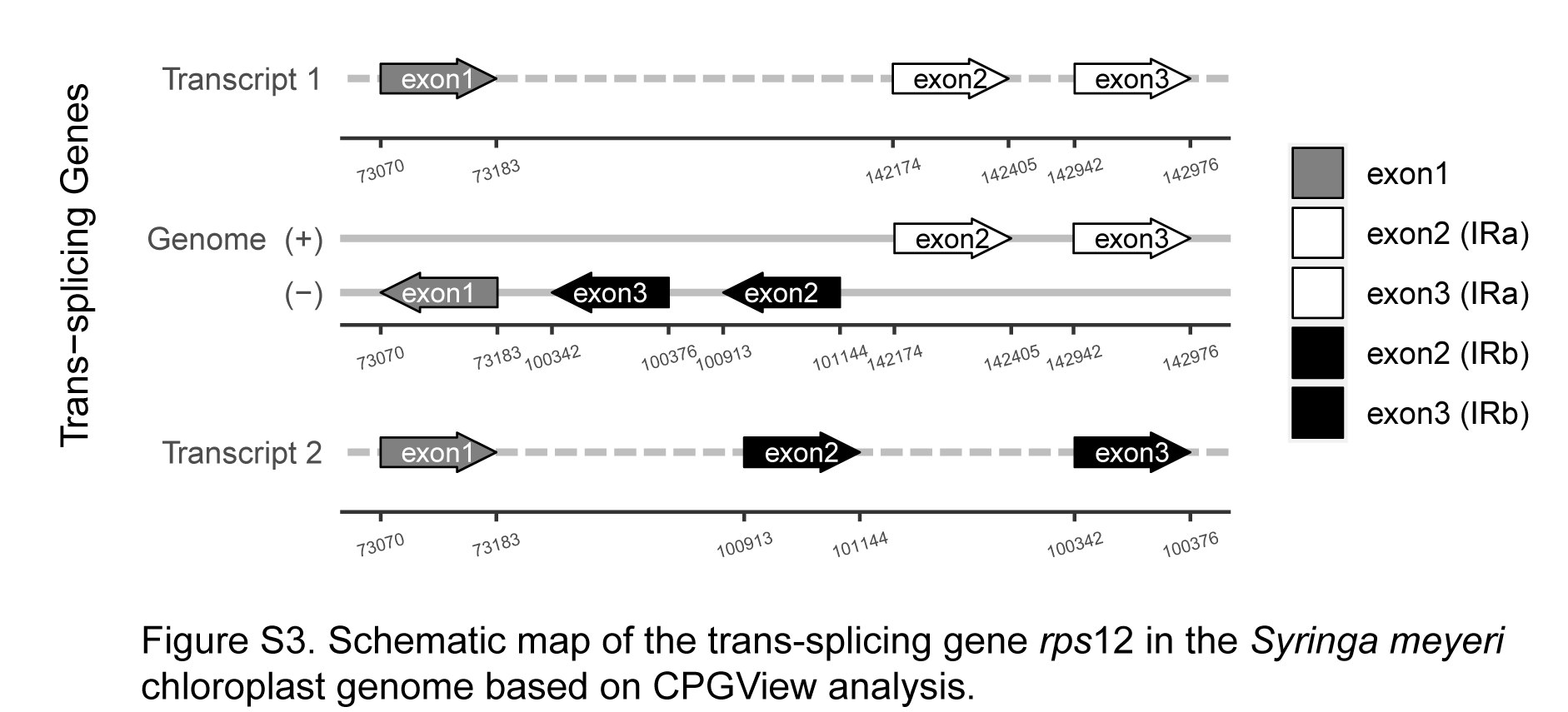

Supplement: Supplementary Material Figure S3.tif [file TMDN_A_2512866_SM7301.tif]

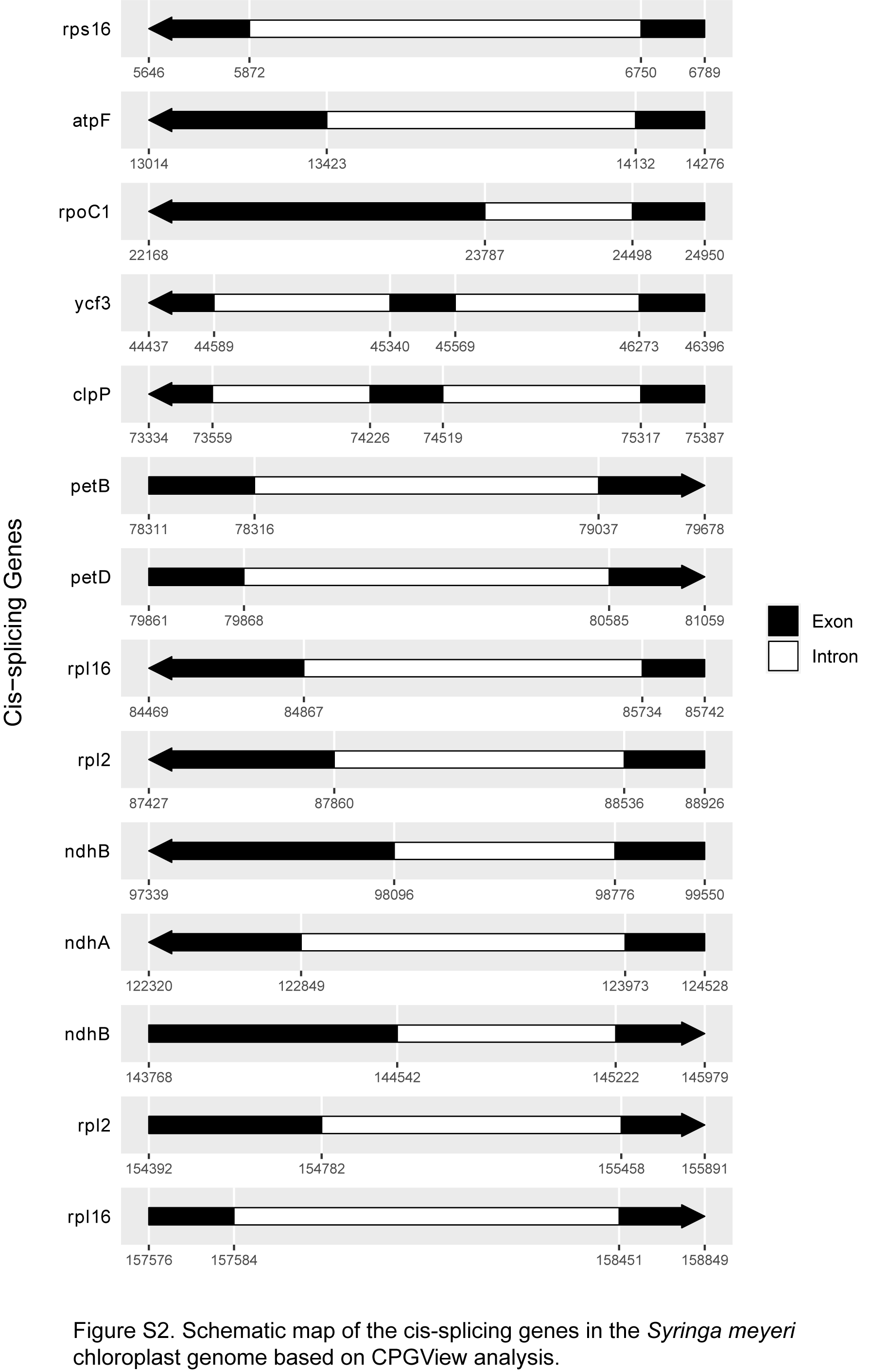

Supplement: Supplementary Material Figure S2.tif [file TMDN_A_2512866_SM7300.tif]

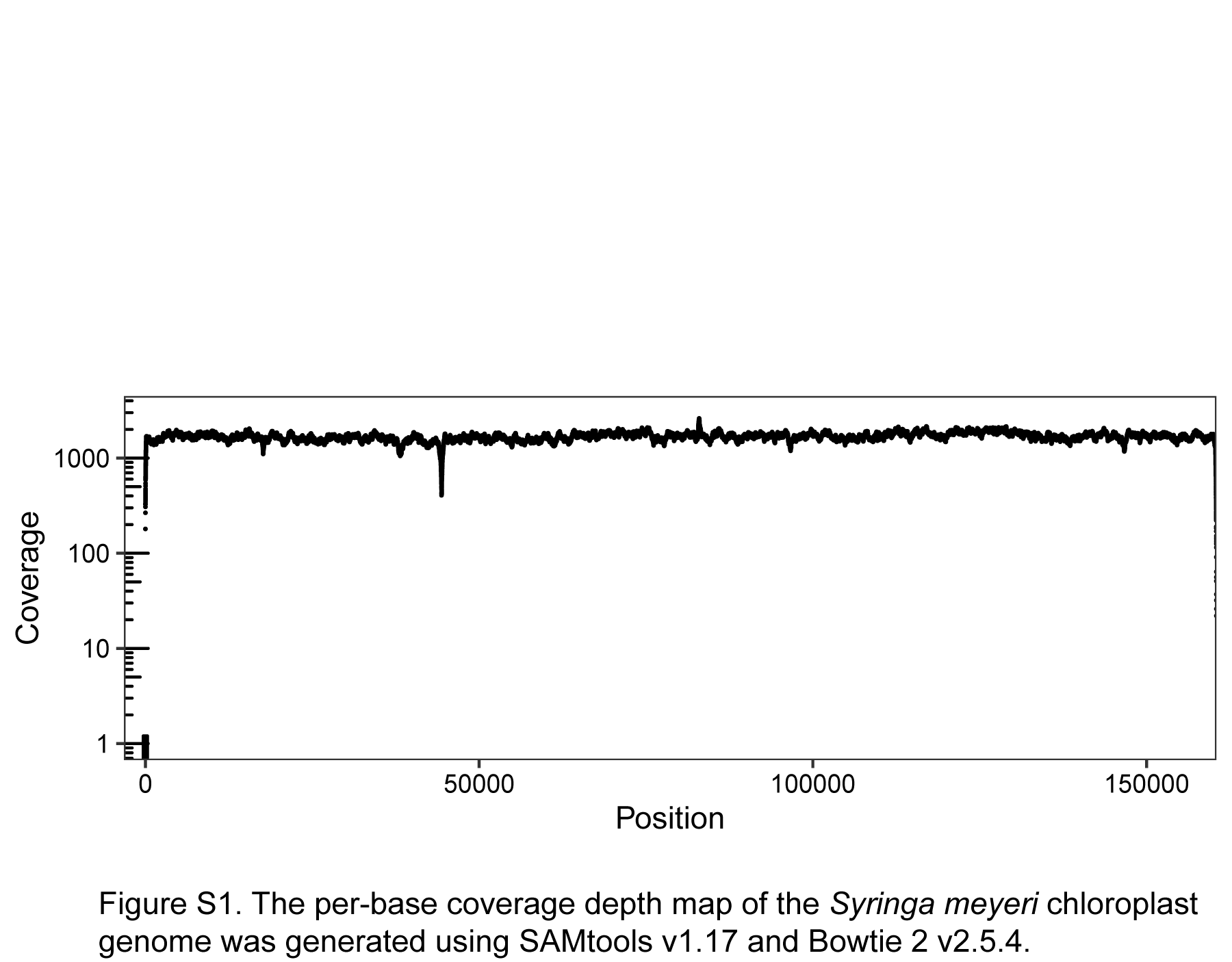

Supplement: Supplementary Material Figure S1.tif [file TMDN_A_2512866_SM7299.tif]
